# Comparative analysis of two modified TLICS systems in guiding surgical decision-making for thoracolumbar fractures

**DOI:** 10.3389/fsurg.2026.1855503

**Published:** 2026-06-26

**Authors:** Han Zhang, Junwei Feng, Bing Wu, Jiayi Dou, Jiale Zhang, Huibin Luo, Binbin Tang, Chen Chen, Liqiang Dong, Zhongcheng An, Tingyuan Lai

**Affiliations:** 1 The Second School of Clinical Medicine of Zhejiang Chinese Medical University, Hangzhou, Zhejiang, China; 2 Wangjing Hospital, China Academy of Chinese Medical Sciences, Beijing, China; 3 Department of Orthopedics II, The Second Affiliated Hospital of Zhejiang Chinese Medical University, Hangzhou, Zhejiang, China; 4 Spine Center, Xinhua Hospital, Shanghai Jiaotong University School of Medicine, Shanghai, China

**Keywords:** CN-mTLICS, intervertebral disc injury, NmTLICS, surgical decision-making, thoracolumbar fractures, TLICS

## Abstract

**Objective:**

To compare the Novel Modified Thoracolumbar Injury Classification System (NmTLICS) and the China-modified Thoracolumbar Injury Classification System (CN-mTLICS) in guiding surgical decision-making for thoracolumbar fractures.

**Methods:**

We retrospectively analyzed the complete imaging data of 101 patients with single-level thoracolumbar fractures admitted between January 2021 and December 2023. Two independent observers scored the patients using the traditional TLICS, NmTLICS, and CN-mTLICS systems. Using the actual clinical treatment decision as the “gold standard,” inter-observer agreement was assessed with Cohen's Kappa coefficient. Discrimination was evaluated by the area under the ROC curve (AUC) with pairwise DeLong comparison; classification accuracy among the three related systems was compared with a global Cochran's *Q*-test followed by *post hoc* McNemar tests with Bonferroni correction; and model calibration and clinical utility were assessed by calibration analysis and decision curve analysis. Discrepant cases between the two modified systems were further analyzed.

**Results:**

All three scoring systems demonstrated excellent inter-observer agreement, with NmTLICS (*κ* = 0.896) and CN-mTLICS (*κ* = 0.866) outperforming traditional TLICS (*κ* = 0.849). Regarding diagnostic efficacy, both NmTLICS and CN-mTLICS showed significantly higher sensitivity and NPV than traditional TLICS (*P* < 0.01). They successfully identified 17 and 16 patients, respectively, who were recommended for conservative treatment by TLICS but actually required surgery. There was no statistically significant difference in specificity compared with TLICS (*P* > 0.05). Discrepancy analysis revealed significant clinical complementarity between the two modified systems: NmTLICS detected 6 cases of severe vertebral collapse (>50%) missed by CN-mTLICS, while CN-mTLICS identified 5 cases of severe intervertebral disc injury missed by NmTLICS.

**Conclusion:**

Both NmTLICS and CN-mTLICS reduced the under-triage of severe burst fractures by TLICS, mainly through higher sensitivity and negative predictive value without a significant loss of specificity, and they address complementary dimensions (fracture morphology and intervertebral disc injury, respectively). As these are exploratory single-center findings based on treatment decisions rather than long-term outcomes, prospective multicenter validation is needed before routine clinical application.

## 1 Introduction

Thoracolumbar fractures are the most common type of spinal trauma, accounting for over 50% of all spinal injuries (1). Owing to the unique anatomy and biomechanical characteristics of this region, improper diagnosis and treatment may expose patients to severe complications such as chronic back pain, secondary kyphosis, and even delayed neurological deficits (2). Therefore, establishing a reliable injury classification and assessment system is crucial for optimizing clinical decisions and improving patient prognosis.

Since its inception, the Thoracolumbar Injury Classification and Severity Score (TLICS) has played a significant role in surgical decision-making. However, as clinical practice has deepened, limitations have emerged. For patients with normal neurological function and an intact posterior ligamentous complex (PLC) but a severe burst fracture, the TLICS score is only 2 points, suggesting conservative treatment. This recommendation can lead to treatment failure or the need for late revision surgery (3–5). Because TLICS dichotomizes fractures into stable and unstable categories, it assigns a score of <4 to burst fractures despite considerable variation in vertebral morphology and canal encroachment, thereby severely underestimating the potential biomechanical instability of such injuries (6). Consequently, some patients who require surgery miss the optimal intervention window, substantially increasing the risk of long-term secondary spinal stenosis and intractable pain (7). To optimize the clinical applicability of TLICS, various modifications have been proposed. Joseph et al. (8) addressed the deficiencies in morphological assessment by proposing the Novel Modified Thoracolumbar Injury Classification System (NmTLICS), which increases the scoring weight for severe vertebral compression and spinal canal stenosis to enhance sensitivity to severe fracture morphology. With advances in spinal biomechanics, researchers have found that the integrity of the intervertebral disc and PLC is critical for spinal stability (9). On this basis, Chinese scholars Lu et al. (10) modified TLICS by innovatively incorporating “intervertebral disc injury” and correcting the scoring weight of the PLC, proposing the China-modified Thoracolumbar Injury Classification System (CN-mTLICS).

Although both modified systems have demonstrated good reliability and reproducibility in previous studies (8, 11), direct comparative research on their guidance of surgical decision-making is currently lacking. Therefore, this study aimed to directly compare NmTLICS and CN-mTLICS in guiding clinical surgical decisions for thoracolumbar fractures, thereby providing evidence-based support for precise clinical diagnosis and treatment.

## 2 Materials and methods

### 2.1 Study design and population

This single-center retrospective study included 101 consecutive patients with single-level thoracolumbar fractures and complete preoperative imaging data who were admitted between January 2021 and December 2023. Preoperative imaging comprised thoracolumbar radiographs, CT with 3D reconstruction, and MRI. All imaging datasets were anonymized and contained no information or annotations related to fracture classification. The study was approved by the hospital's Ethics Committee, and the requirement for informed consent was waived owing to the retrospective design.

### 2.2 Inclusion criteria

Patients were eligible if they had a traumatic single-level thoracolumbar fracture confirmed by initial imaging and a complete clinical and imaging dataset that comprised preoperative anteroposterior and lateral radiographs of the thoracolumbar spine, computed tomography (CT) with three-dimensional vertebral reconstruction, and magnetic resonance imaging (MRI) of the injured segment.

### 2.3 Exclusion criteria

Patients were excluded if they presented with any of the following: multilevel or old thoracolumbar fractures involving two or more vertebral levels; non-traumatic fractures, including pathological fractures related to spinal tumors, infection, or osteoporosis; pre-existing neurological impairment before the fracture event; concomitant pelvic or lower-limb fractures requiring surgical intervention; or incomplete clinical or imaging datasets, such as missing preoperative MRI or CT scans.

### 2.4 Imaging protocol and definitions

Fracture morphology was assessed by analyzing thoracolumbar radiographs, computed tomography (CT) 3D reconstructions, and magnetic resonance imaging (MRI). The percentage of height loss was calculated by measuring the minimum height of the fractured vertebral body on sagittal CT images and dividing it by the height of the normal vertebral body above the injury. The percentage of spinal canal stenosis was calculated by measuring the narrowest canal distance at the fracture level and dividing it by the normal canal width above the injury. The MRI diagnostic criteria for PLC injury were defined on sagittal T2-weighted fat-suppressed sequences; discontinuity of the ligament or high signal intensity on these sequences suggested PLC injury (12). The MRI grading system for intervertebral disc injury (13) classified the adjacent disc into four grades (0–3) on the basis of morphological and signal changes. Grade 0: high signal on T2-weighted imaging (T2WI) with preserved morphology. Grade 1: represents disc edema, characterized by high signal intensity on T2-weighted imaging (T2WI) with preserved disc morphology. Grade 2: disc rupture, iso- or hyperintense on T1WI, hypointense on T2WI, with surrounding hyperintensity. Grade 3: disc invasion into the vertebral body, manifesting as annular tears or herniation into the vertebral body, iso- or hyperintense on T1WI. Neurological deficits were assessed with the ASIA impairment scale (14).

### 2.5 Scoring methodology

Two orthopedic attending physicians and one senior chief physician were invited to study the TLICS, NmTLICS, and CN-mTLICS scoring systems and were subsequently tested. After passing the evaluation, the two attending physicians independently scored the imaging data of the 101 patients. To minimize bias, the raters were blinded to the patients' subsequent treatment and clinical outcomes. When the scores and treatment recommendations of the two surgeons agreed, the result was accepted; when they disagreed, the case was referred to the chief surgeon for a final decision. This process helped reduce errors by junior physicians in assessing complex cases, thereby improving the consistency and accuracy of the scoring results.

TLICS (Table 1) (15) comprises three components: fracture morphology, neurological status, and PLC integrity. A cumulative score <4 suggests non-surgical treatment; a score of 4 allows either non-surgical or surgical treatment based on patient preference or surgeon discretion; a score >4 suggests surgical treatment.

**Table 1:** Thoracolumbar injury classification system (TLICS).

Injury Type	Features	Score
Morphological Injury	Compression	1
	Burst fracture	2
	Displacement/rotation	3
	Distraction	4
Neurological Impairment	No neurological deficit	0
	Nerve root injury	2
	Complete spinal cord/conus medullaris injury	2
	Incomplete spinal cord/conus medullaris injury	3
	Cauda equina injury	3
Posterior Ligamentous Complex (PLC) Injury	Intact	0
	Suspected injury	1
	Injury	2

Total Score (T): T < 4, conservative treatment; T = 4, conservative or surgical treatment; T > 4, surgical treatment.

NmTLICS (Table 2) (8) is a novel modified TLICS system. Its assessment of injury type, neurological status, and PLC injury is consistent with the original TLICS. The modification lies in adding an extra 2 points when vertebral height loss is >50% and/or spinal canal stenosis is >50%. A total score (T) < 4 suggests conservative treatment; *T* = 4 allows a conservative or surgical choice; *T* > 4 recommends surgery.

**Table 2:** Novel modified thoracolumbar injury classification system (NmTLICS).

Injury Type	Features	Score
Morphological Injury	Compression	1
	Burst fracture	2
	Displacement/rotation	3
	Distraction	4
Neurological Impairment	No neurological deficit	0
	Nerve root injury	2
	Complete spinal cord/conus medullaris injury	2
	Incomplete spinal cord/conus medullaris injury	3
	Cauda equina injury	3
Posterior Ligamentous Complex (PLC) Injury	Intact	0
	Suspected injury	1
	Injury	2
Proposed fracture modiﬁer	Spinal canal stenosis >50% and/or vertebral body height loss >50%	2

Total Score (T): T < 4, conservative treatment; T = 4, conservative or surgical treatment; T > 4, surgical treatment.

CN-mTLICS (Table 3) (11) is another TLICS-based modification that adds an “intervertebral disc injury status” subcategory and adjusts the scoring weight of the “PLC integrity” subcategory. The total score is 11 points. “Intervertebral disc injury status” follows the Sander classification: no injury (Grade 0, 0 points), mild injury (Grade 1, 1 point), and moderate-to-severe injury (Grades 2–3, 2 points). “PLC integrity” is scored as no injury (0 points), suspected injury (1 point), or definite injury (2 points). Fracture morphology and neurological scores are consistent with the original TLICS. A score <4 suggests conservative treatment; a score of 4 allows conservative or surgical treatment depending on the patient's systemic condition and quality of life; a score >4 suggests surgical treatment.

**Table 3:** China modified thoracolumbar injury classification and severity score system (CN-mTLICS).

Injury Type	Features	Score
Morphological Injury	Compression	1
	Burst fracture	2
	Displacement/rotation	3
	Distraction	4
Neurological Impairment	No neurological deficit	0
	Nerve root injury	2
	Complete spinal cord/conus medullaris injury	2
	Incomplete spinal cord/conus medullaris injury	3
	Cauda equina injury	3
Posterior Ligamentous Complex (PLC) Injury	Intact	0
	Suspected injury	1
	Injury	2
Intervertebral Disc Injury Status	No injury	0
	Mild injury	1
	Moderate-to-severe injury	2

Total Score (T): T < 4, conservative treatment; T = 4, conservative or surgical treatment; T > 4, surgical treatment.

### 2.6 Statistical analysis

Continuous variables were summarized as mean ± standard deviation. Inter-observer agreement between the two independent raters was assessed for each scoring system with the linearly weighted Cohen's kappa coefficient, interpreted as poor (<0.20), fair (0.21–0.40), moderate (0.41–0.60), good (0.61–0.80), or excellent (0.81–1.00). Taking the actual clinical treatment decision (surgery vs. non-surgery) as the reference standard, the sensitivity, specificity, accuracy, and positive and negative predictive values (PPV and NPV) of each system were calculated at the predefined surgical threshold of a total score ≥4, with 95% confidence intervals (CIs) for proportions obtained by the Wilson score method. Discrimination was quantified by the area under the receiver operating characteristic curve (AUC) with DeLong 95% CIs, and the three correlated AUCs were compared pairwise using the DeLong test with Bonferroni correction. Because three related scoring systems were compared, the overall difference in classification accuracy was first examined with a global Cochran's *Q-*test; when significant, *post hoc* pairwise comparisons were performed with the exact (binomial) McNemar test using Bonferroni correction (adjusted significance threshold *α* = 0.05/3 = 0.0167). Model calibration was assessed by mapping each total score to a predicted probability of surgery with univariable logistic regression, summarized by calibration plots and an optimism-corrected calibration slope from bootstrap internal validation (500 resamples), and clinical utility was evaluated by decision curve analysis across a range of threshold probabilities. The incremental predictive value of the two modified systems relative to TLICS was further quantified by the categorical net reclassification improvement (NRI) and the integrated discrimination improvement (IDI), with 95% CIs obtained from 2000 bootstrap resamples. This three-tiered evaluation of discrimination, calibration, and clinical utility is consistent with recent validation studies of thoracolumbar injury classification algorithms (8). A *P*-value < 0.05 was considered statistically significant. Statistical analyses were also performed on factors causing inconsistencies between the two scoring systems to explore reasons affecting classification, scoring, and surgical decision-making. Baseline characteristics were compared between the surgical and non-surgical groups: continuous variables (age and body mass index), which were normally distributed within each group by the Shapiro–Wilk test, were compared with Welch's *t*-test and reported as mean ± SD, and categorical variables were compared with the Pearson *χ*^2^-test or with Fisher's exact test when more than 20% of cells had an expected count below 5 (fracture morphology and injury level). All statistical analyses were performed using IBM SPSS Statistics (IBM Corp., Armonk, NY, USA) and R (R Foundation for Statistical Computing, Vienna, Austria).

## 3 Results

A total of 101 patients with thoracolumbar fractures were included, of whom 74 (73.3%) underwent surgical treatment and 27 (26.7%) were managed non-operatively. The mean age of the cohort was 40.3 ± 7.8 years; 50 patients (49.5%) were male and 51 (50.5%) female, with a mean body mass index of 23.6 ± 3.7 kg/m^2^. The principal injury mechanisms were falls (59 patients, 58.4%) and traffic accidents (42 patients, 41.6%); injuries were concentrated at the thoracolumbar junction, most commonly at L1 (38 patients, 37.6%) and T12 (37 patients, 36.6%). The surgical and non-surgical groups did not differ significantly in age, sex, body mass index, mechanism of injury, or injury level (all *P* > 0.05), indicating comparable baseline characteristics (Table 4). With respect to injury-severity variables, the surgical group had significantly higher proportions of neurological deficit, vertebral compression or canal compromise >50%, posterior ligamentous complex injury (indeterminate or disrupted), higher disc-injury (Sander) grades, and high-energy fracture morphologies (burst, distraction, and translation/rotation) than the non-surgical group (*P* values in Table 4), consistent with the clinical expectation that these features drive the decision to operate. For inter-observer agreement, blind assessment by two independent observers showed excellent consistency for all three scoring systems. The weighted Kappa coefficient for traditional TLICS was 0.849 (*P* < .001), with a complete agreement rate of 87.1%. Reliability improved slightly over TLICS after the introduction of the modified variables. NmTLICS demonstrated the highest consistency (Kappa = 0.896, *P* < 0.001), with a complete agreement rate of 92.1%. CN-mTLICS also maintained very high consistency (Kappa = 0.866, *P* < 0.001).

**Table 4:** Baseline characteristics of the cohort, stratified by management (surgical vs. non-surgical).

Characteristic	Conservative (*n* = 27)	Surgical (*n* = 74)	Total (*n* = 101)	*P* value
Age, years	38.0 ± 7.8	41.2 ± 7.6	40.3 ± 7.8	0.076
Sex, *n* (%)				0.539
Male	12 (44.4)	38 (51.4)	50 (49.5)	
Female	15 (55.6)	36 (48.6)	51 (50.5)	
Body mass index, kg/m²	23.0 ± 3.3	23.8 ± 3.9	23.6 ± 3.7	0.305
Mechanism of injury, *n* (%)				0.054
Fall	20 (74.1)	39 (52.7)	59 (58.4)	
Traffic accident	7 (25.9)	35 (47.3)	42 (41.6)	
Injury level, *n* (%)				0.664
T11	2 (7.4)	8 (10.8)	10 (9.9)	
T12	12 (44.4)	25 (33.8)	37 (36.6)	
L1	8 (29.6)	30 (40.5)	38 (37.6)	
L2	5 (18.5)	11 (14.9)	16 (15.8)	
Neurological status, *n* (%)				0.006
Intact (ASIA E)	25 (92.6)	44 (59.5)	69 (68.3)	
Nerve root injury	2 (7.4)	21 (28.4)	23 (22.8)	
Incomplete spinal cord injury	0 (0.0)	9 (12.2)	9 (8.9)	
Vertebral compression or canal compromise >50%, *n* (%)	6 (22.2)	54 (73.0)	60 (59.4)	<0.001
Fracture morphology, *n* (%)				<0.001
Compression	12 (44.4)	0 (0.0)	12 (11.9)	
Burst	15 (55.6)	57 (77.0)	72 (71.3)	
Translation/rotation	0 (0.0)	6 (8.1)	6 (5.9)	
Distraction	0 (0.0)	11 (14.9)	11 (10.9)	
PLC status, *n* (%)				0.012
Intact	23 (85.2)	39 (52.7)	62 (61.4)	
Indeterminate	2 (7.4)	17 (23.0)	19 (18.8)	
Disrupted	2 (7.4)	18 (24.3)	20 (19.8)	
Disc injury (Sander grade), *n* (%)				<0.001
Grade 0	19 (70.4)	6 (8.1)	25 (24.8)	
Grade 1	5 (18.5)	30 (40.5)	35 (34.7)	
Grade 2	3 (11.1)	38 (51.4)	41 (40.6)	

Data are mean ± SD or *n* (%). Continuous variables were compared with Welch's *t*-test (both age and body mass index were normally distributed within each group by the Shapiro–Wilk test). Categorical variables were compared with the Pearson *χ*^2^-test, or with Fisher's exact test when more than 20% of cells had an expected count below 5 (fracture morphology and injury level). ASIA, American Spinal Injury Association; BMI, body mass index; PLC, posterior ligamentous complex.

The overall difference in classification accuracy among the three systems was statistically significant (Cochran's Q = 16.23, df = 2, *P* < 0.001). *post hoc* pairwise comparisons using the exact McNemar test with Bonferroni correction (adjusted threshold *α* = 0.05/3 = 0.0167) showed that both modified systems were significantly more accurate than traditional TLICS: in the TLICS–NmTLICS comparison, 17 patients misclassified by TLICS were correctly reclassified by NmTLICS vs. only 3 in the opposite direction (*P* = 0.003), and an analogous pattern was observed for CN-mTLICS vs. TLICS (17 vs. 2; *P* < 0.001). The two modified systems did not differ significantly from each other (*P* = 1.00).

Regarding sensitivity, both modified systems were significantly better than traditional TLICS (*p* < 0.01). Notably, in the comparison between TLICS and NmTLICS, the sensitivity of NmTLICS was significantly higher (*p* < 0.01). NmTLICS successfully identified 17 surgical patients missed by TLICS without missing any patient detected by TLICS. CN-mTLICS showed a similar advantage, identifying 16 patients missed by TLICS, also without missing any cases detected by TLICS; its sensitivity was significantly superior to that of TLICS (*p* < 0.01). Comparison of the two modified systems showed no statistically significant difference in sensitivity between NmTLICS and CN-mTLICS. Discrepancy analysis showed that NmTLICS detected 6 patients missed by CN-mTLICS, whereas CN-mTLICS detected 5 patients missed by NmTLICS, indicating no difference in sensitivity performance.

Regarding specificity, neither modified system differed significantly from traditional TLICS (*p* > 0.05), indicating that the modified systems maintained comparable specificity while significantly improving sensitivity. In the TLICS vs. NmTLICS comparison, only 3 patients correctly identified for conservative treatment by TLICS were misjudged as requiring surgery by NmTLICS, a difference that was not statistically significant (*p* = 0.25). Similarly, the specificity difference between CN-mTLICS and TLICS was not significant. Discrepancy analysis showed that CN-mTLICS produced 2 new false positives but corrected 1 false positive from TLICS. The two modified systems were highly consistent in specificity, with only 1 discordant interpretation.

Diagnostic performance at the predefined surgical threshold (total score ≥4) is summarized in Table 5. Sensitivity was 64.9% (95% CI 53.5–74.8), 87.8% (78.5–93.5), and 86.5% (76.9–92.5) for TLICS, NmTLICS, and CN-mTLICS, respectively; specificity was 81.5% (63.3–91.8), 70.4% (51.5–84.1), and 77.8% (59.2–89.4); and overall accuracy was 69.3%, 83.2%, and 84.2%. The most marked improvement was in the negative predictive value (NPV), which rose from 45.8% with TLICS to 67.9% with NmTLICS and 67.7% with CN-mTLICS, reflecting a substantial reduction in missed surgical cases.

**Table 5:** Diagnostic performance and statistical comparison of the three scoring systems in predicting surgical intervention.

**System**	**AUC (95% CI)**	**Sensitivity, % (95% CI)**	**Specificity, % (95% CI)**	**PPV, %**	**NPV, %**	**Accuracy, %**
TLICS	0.815 (0.724–0.905)	64.9 (53.5–74.8)	81.5 (63.3–91.8)	90.6	45.8	69.3
NmTLICS	0.856 (0.771–0.941)	87.8 (78.5–93.5)	70.4 (51.5–84.1)	89.0	67.9	83.2
CN-mTLICS	0.887 (0.808–0.966)	86.5 (76.9–92.5)	77.8 (59.2–89.4)	91.4	67.7	84.2

AUC, area under the receiver operating characteristic curve; PPV, positive predictive value; NPV, negative predictive value. 95% confidence intervals for proportions were calculated by the Wilson method and for the AUC by the DeLong method. Pairwise AUC comparisons used the DeLong test, and classification-accuracy comparisons used a global Cochran's *Q*-test followed by *post-hoc* McNemar tests; all multiplicity adjustments used the Bonferroni method.

Sensitivity reflects the scoring system's ability to “detect” patients requiring surgery; Specificity measures the system's capacity to “exclude” non-surgical patients; Accuracy represents the overall correctness of predictions; Positive Predictive Value (PPV) reflects the reliability of surgical indications; and Negative Predictive Value (NPV) indicates the probability that patients actually do not require surgery.

The positive predictive value (PPV) remained high and similar across systems (90.6%, 89.0%, and 91.4% for TLICS, NmTLICS, and CN-mTLICS, respectively), indicating that the gains in sensitivity and NPV were achieved without a meaningful increase in false-positive surgical recommendations. The two modified systems did not differ significantly from each other in PPV or NPV.

**Figure 1:**
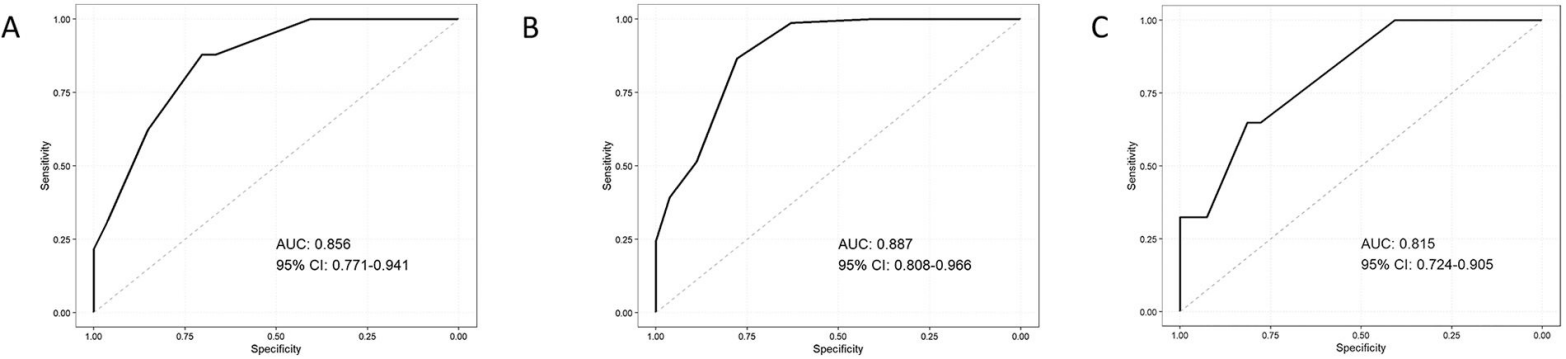
Receiver operating characteristic (ROC) curve analysis of the three scoring systems for predicting surgical decision-making. Panels **(A)**, **(B)**, and **(C)** represent TLICS, NmTLICS, and CN-mTLICS, respectively. The areas under the curve for TLICS, NmTLICS, and CN-mTLICS were 0.887, 0.856, and 0.815, with corresponding 95% confidence intervals of 0.808–0.966, 0.771–0.941, and 0.724–0.905, respectively, indicating good discriminatory performance for all three scoring systems. ROC, receiver operating characteristic; AUC, area under the curve; CI, confidence interval.

ROC curve analysis showed that all three systems had good discriminative ability (AUC > 0.8) (Figure 1). The AUC was 0.815 (95% CI 0.724–0.905) for TLICS, 0.856 (0.771–0.941) for NmTLICS, and 0.887 (0.808–0.966) for CN-mTLICS. In pairwise DeLong comparisons, both modified systems showed numerically higher discrimination than TLICS (ΔAUC = 0.041, *P* = 0.039 for NmTLICS; ΔAUC = 0.072, *P* = 0.018 for CN-mTLICS); however, neither difference remained statistically significant after Bonferroni correction (adjusted *P* = 0.116 and 0.055, respectively), and the two modified systems did not differ from each other (*P* = 0.106).

**Figure 2:**
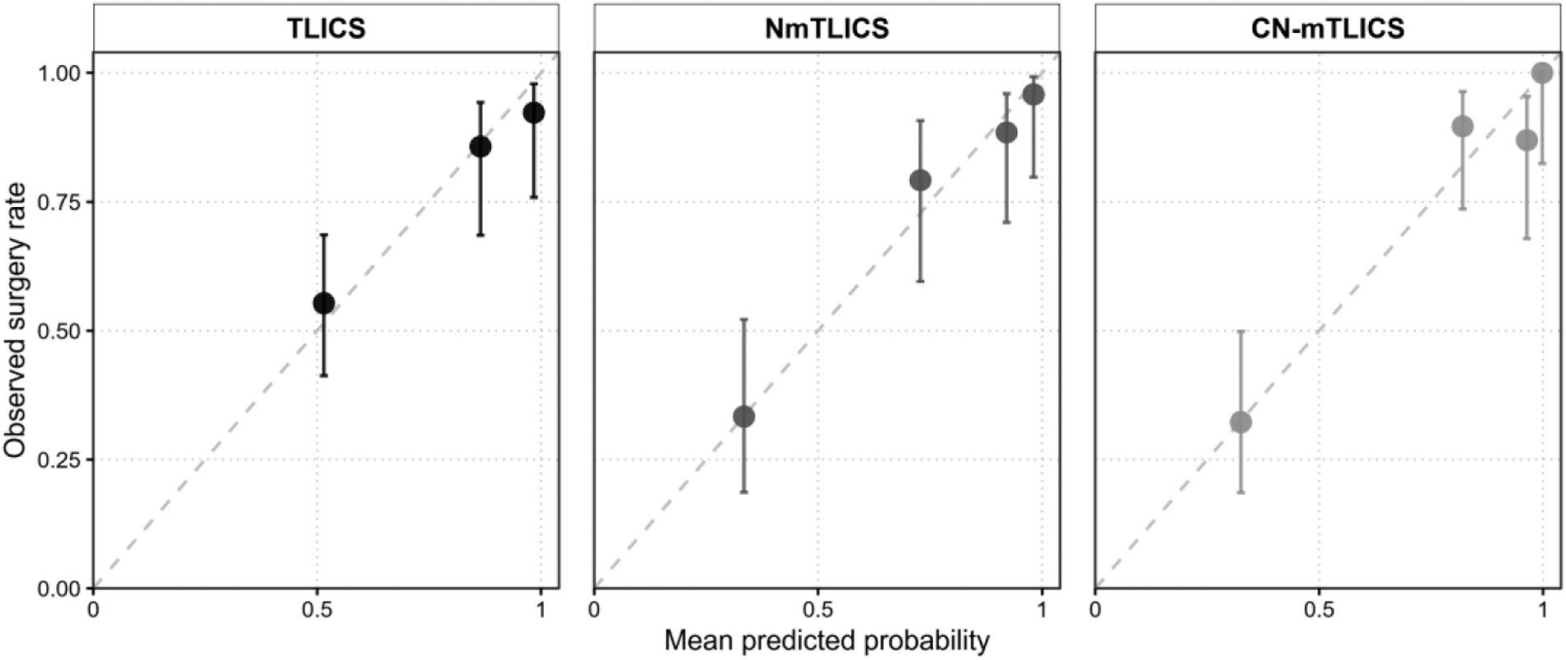
Calibration plots of the predicted probability of surgery and the observed surgery rate for the three scoring systems. The *x*-axis represents the mean predicted probability of surgery, and the *y*-axis represents the observed surgery rate. The dashed diagonal line indicates perfect calibration. Points and error bars represent the observed surgery rate and corresponding 95% confidence intervals within each group, respectively. Overall, the predicted probabilities derived from TLICS, NmTLICS, and CN-mTLICS showed good agreement with the observed surgery rates.

**Figure 3:**
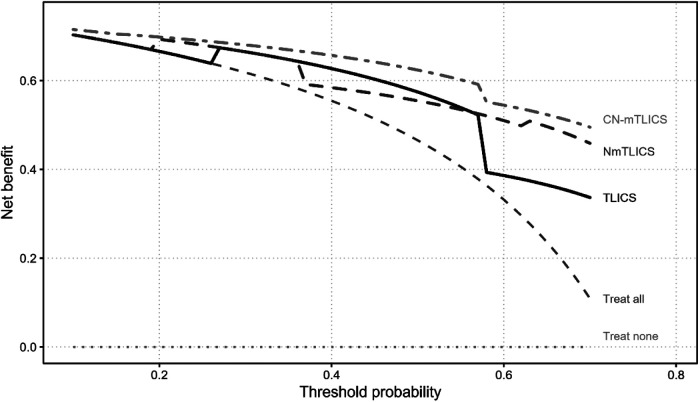
Decision curve analysis of the three scoring systems. The *x*-axis represents the threshold probability, and the *y*-axis represents the net benefit. The curves for TLICS, NmTLICS, and CN-mTLICS indicate the clinical net benefit of each scoring system for surgical decision-making across different threshold probabilities. The “treat-all” and “treat-none” lines represent the reference strategies in which all patients or no patients receive surgical treatment, respectively. Overall, all three scoring systems showed greater net benefit than the reference strategies across most threshold probabilities.

All three score-to-probability models were well calibrated. Bootstrap internal validation (500 resamples) yielded optimism-corrected calibration slopes close to 1.0 (1.02, 1.00, and 0.98 for TLICS, NmTLICS, and CN-mTLICS, respectively) with a negligible reduction in the c-statistic (≤0.002), and the calibration plots showed good agreement between predicted and observed surgical rates (Figure 2). On decision curve analysis, CN-mTLICS provided the highest net benefit across the clinically relevant range of threshold probabilities, exceeding TLICS, NmTLICS, and the treat-all and treat-none reference strategies (for example, a net benefit of 0.680 vs. 0.665 vs. 0.618 at a threshold probability of 0.3; Figure 3).

**Figure 4:**
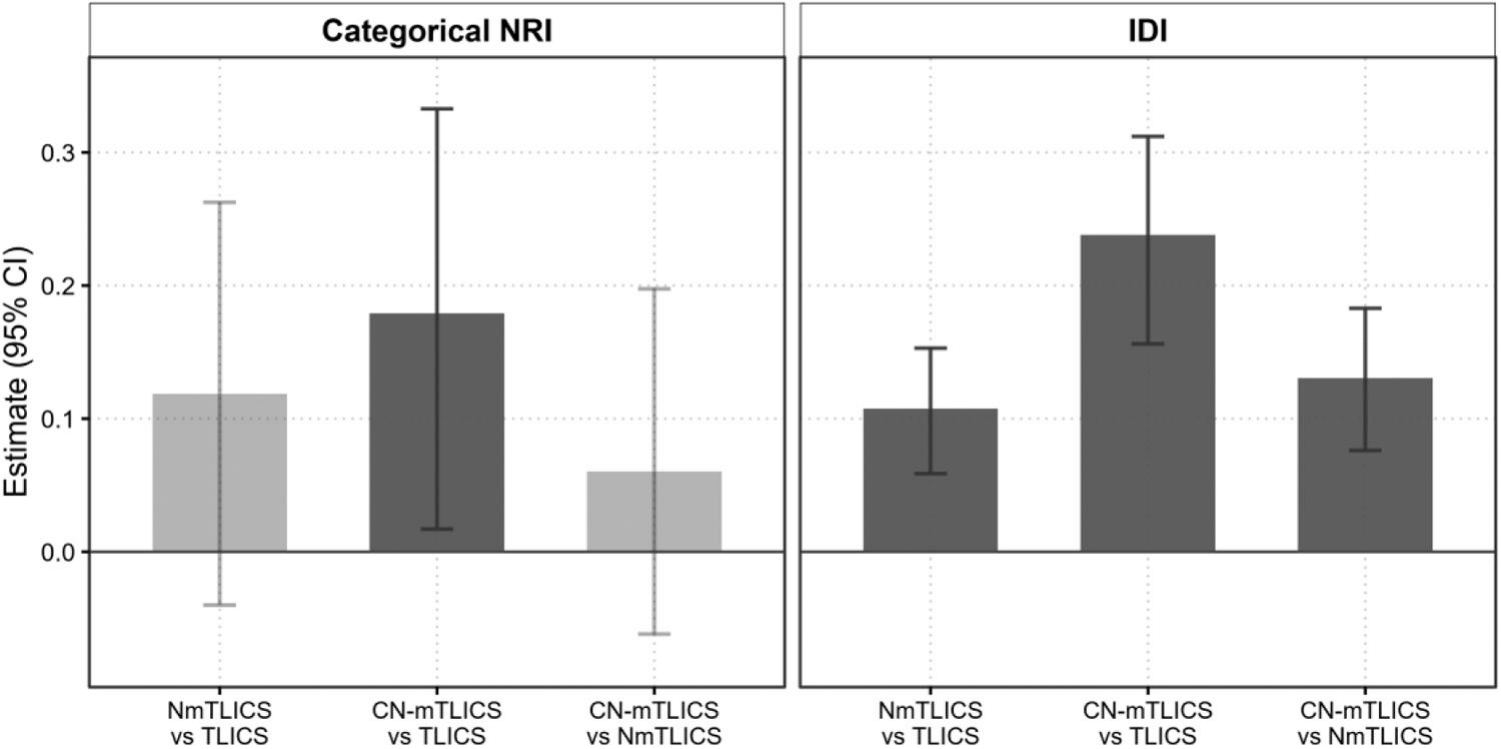
Reclassification analysis of the two modified systems relative to the reference systems. The left panel shows the categorical net reclassification improvement (NRI), based on the binary surgical decision (total score ≥4), and the right panel shows the integrated discrimination improvement (IDI), for NmTLICS vs. TLICS, CN-mTLICS vs. TLICS, and CN-mTLICS vs. NmTLICS. Bars represent point estimates and error bars represent 95% confidence intervals derived from 2000 bootstrap resamples. NRI, net reclassification improvement; IDI, integrated discrimination improvement.

Relative to TLICS, both modified systems improved risk reclassification (Figure 4). The IDI was 0.107 (95% CI, 0.059–0.153) for NmTLICS and 0.238 (95% CI, 0.156–0.312) for CN-mTLICS, indicating a significant gain in discrimination slope for both systems. The categorical NRI, based on the binary surgical decision (total score ≥4), was significant for CN-mTLICS vs. TLICS (0.179; 95% CI, 0.017–0.333) but did not reach significance for NmTLICS vs. TLICS (0.119; 95% CI, −0.040 to 0.263). Compared with NmTLICS, CN-mTLICS showed a further improvement in IDI (0.130; 95% CI, 0.076–0.183), although the categorical NRI did not differ significantly (0.061; 95% CI, −0.062 to 0.197).

Analysis of patients in the diagnostic “gray zone” (total score = 4) showed that the large majority ultimately underwent surgery under every system: 24 of 27 (88.9%) for TLICS, 19 of 23 (82.6%) for NmTLICS, and 26 of 29 (89.7%) for CN-mTLICS. Because the two modified systems reclassified partially non-overlapping subsets into the surgical range—NmTLICS capturing cases of severe vertebral collapse and CN-mTLICS capturing cases of severe disc injury—these gray-zone findings support a complementary rather than redundant relationship between the two systems.

**Figure 5:**
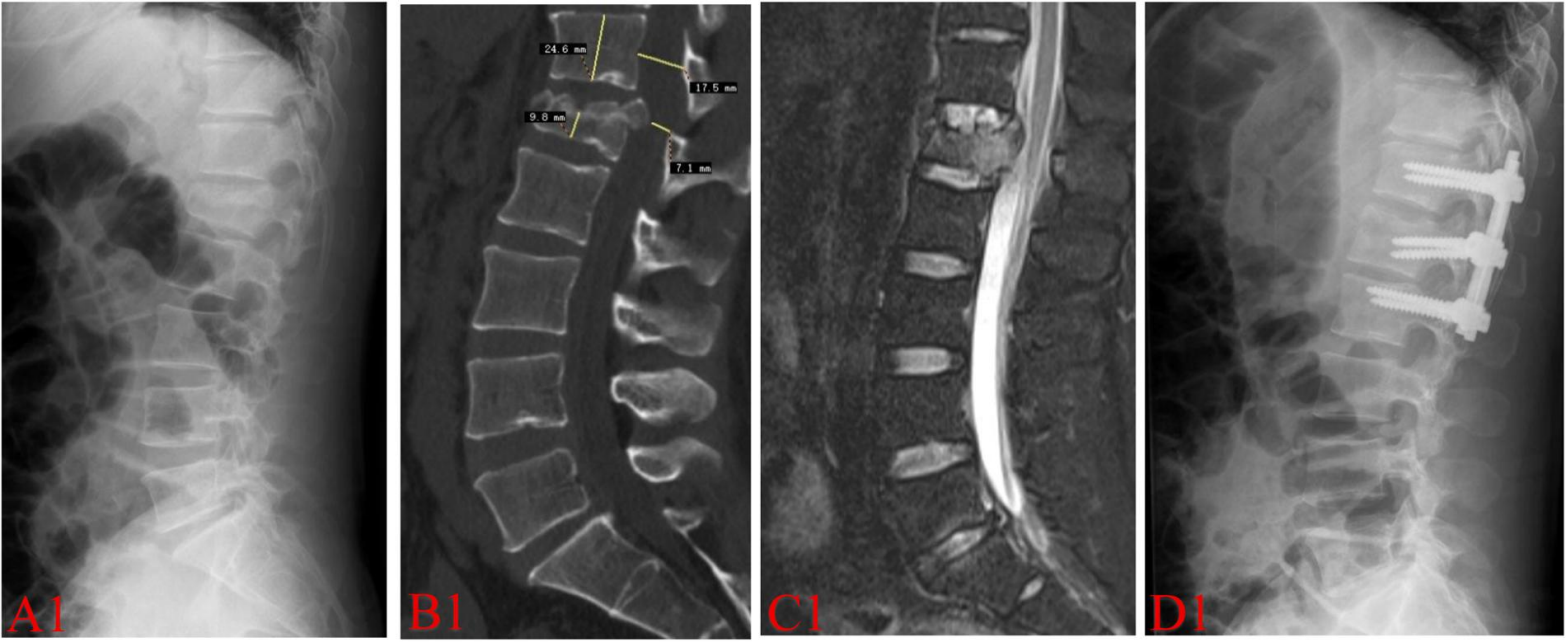
Representative case 1. A 35-year-old male presented following a motor vehicle accident. CT imaging (B1) revealed an L1 burst fracture characterized by severe comminution of both the superior and inferior endplates, vertebral height collapse greater than 50%, and spinal canal encroachment exceeding 50% due to retropulsed bone fragments; the posterior ligamentous complex (PLC) appeared intact, and no neurological deficits were observed. MRI (C1) demonstrated severe intervertebral disc injury. The patient received a TLICS score of 2 (indicating conservative treatment), whereas both NmTLICS and CN-mTLICS assigned a score of 4 (indicating an indeterminate recommendation). Ultimately, the patient underwent posterior spinal fixation from T12 to L2 (D1). In this case, TLICS failed to align with the actual treatment regimen, while NmTLICS and CN-mTLICS successfully predicted the surgical intervention. CT, Computed Tomography; MRI, Magnetic Resonance Imaging; TLICS, Thoracolumbar Injury Classification and Severity Score; NmTLICS, Novel Modified Thoracolumbar Injury Classification System; CN-mTLICS, China-modified Thoracolumbar Injury Classification System.

**Figure 6:**
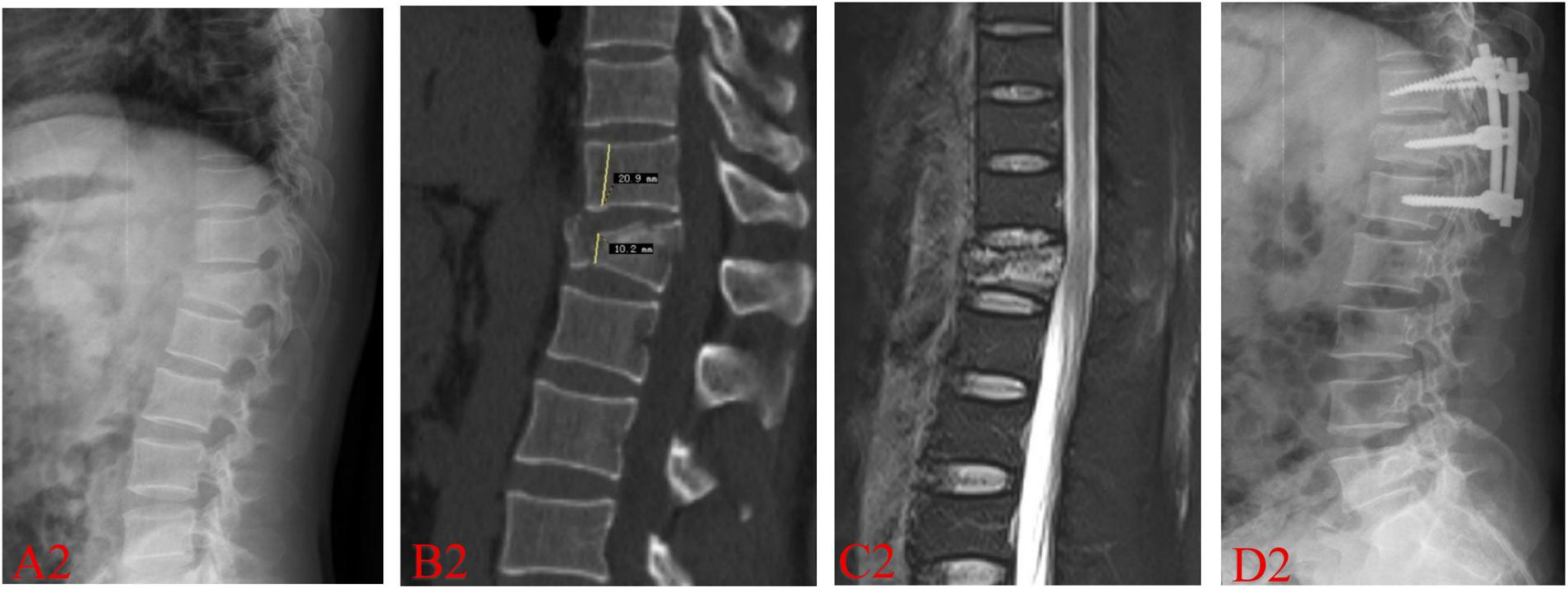
Representative case 2. A 50-year-old male presented following a motor vehicle accident. CT imaging (B2) revealed a T12 burst fracture characterized by severe comminution of both the superior and inferior endplates and vertebral height collapse exceeding 50%, while spinal canal encroachment remained less than 50%. The posterior ligamentous complex (PLC) appeared intact, and no neurological deficits were identified. MRI (C2) indicated mild intervertebral disc injury. The patient received a TLICS score of 2 (indicating conservative treatment), an NmTLICS score of 4 (indicating an indeterminate recommendation), and a CN-mTLICS score of 3 (indicating conservative treatment). Ultimately, the patient underwent posterior spinal fixation from T11 to L1 (D2). In this instance, both TLICS and CN-mTLICS failed to align with the actual treatment regimen, whereas only NmTLICS successfully predicted the surgical intervention. CT, Computed Tomography; MRI, Magnetic Resonance Imaging; TLICS, Thoracolumbar Injury Classification and Severity Score; NmTLICS, Novel Modified Thoracolumbar Injury Classification System; CN-mTLICS, China-modified Thoracolumbar Injury Classification System.

Typical cases are illustrated in Figures 5, 6.

## 4 Discussion

In the management of thoracolumbar fractures, the TLICS system has become a cornerstone of clinical decision-making, pioneering the integration of neurological status into injury assessment (16). Its treatment of vertebral morphology, however, is comparatively coarse: burst fractures of widely differing severity are all assigned the same 2 points. Because of this low morphological weighting, some patients with severe burst fractures are steered toward conservative management and may miss the optimal surgical window. Multiple studies have shown that underestimating the biomechanical instability of such injuries can expose patients to delayed kyphosis, intractable low-back pain, and even neurological deterioration, culminating in failure of conservative treatment (5, 8, 17, 18). NmTLICS and CN-mTLICS were proposed to address precisely this shortcoming, by adding or refining morphological scoring rules.

In the present study, we compared the ability of TLICS, NmTLICS, and CN-mTLICS to predict the treatment actually received by patients with traumatic thoracolumbar fractures. All three systems showed robust discrimination, with AUCs consistently above the 0.8 threshold (19) (TLICS 0.815, NmTLICS 0.856, CN-mTLICS 0.887). Although the two modified systems achieved numerically higher AUCs, these pairwise differences did not remain statistically significant after Bonferroni correction and should therefore be regarded as exploratory. Their advantage emerged most clearly in classification performance: in a global Cochran's *Q*-test followed by Bonferroni-corrected McNemar comparisons, both modified systems classified treatment significantly more accurately than TLICS, driven mainly by gains in sensitivity and negative predictive value for unstable injuries—thereby correcting the tendency of TLICS to overlook some severe burst fractures.

Discrimination alone does not establish clinical usefulness, so we additionally examined calibration and net benefit. All three score-to-probability models were well calibrated, with optimism-corrected calibration slopes close to 1.0, indicating close agreement between predicted and observed surgical probabilities. On decision curve analysis, CN-mTLICS provided the highest net benefit across the clinically relevant range of threshold probabilities, exceeding TLICS, NmTLICS, and the treat-all and treat-none reference strategies. Although the gain in discrimination did not reach significance, the reclassification metrics did: relative to TLICS, the IDI was significantly positive for both NmTLICS and CN-mTLICS (0.107 and 0.238), and CN-mTLICS significantly improved categorical reclassification of the surgical decision (NRI 0.179), indicating that the morphological refinements contribute genuine incremental predictive information rather than merely redistributing borderline cases (Figure 4). These findings suggest that the incremental value of the modified systems, though not statistically significant in discrimination, may translate into a modest gain in decision-making; consistent with the discrimination results, this gain should be interpreted as exploratory pending external validation.

The two modified systems showed a marked “leak-plugging” capacity, recapturing 17 (NmTLICS) and 16 (CN-mTLICS) patients who ultimately underwent surgery but whom TLICS had classified as candidates for conservative treatment. These patients scored only 2 points under TLICS yet reached a total of 4 points under the modified systems—through additional points for substantial vertebral height loss or canal compromise (NmTLICS) or for severe disc injury (CN-mTLICS)—and all proceeded to surgery. Importantly, neither modified system reclassified any patient whom TLICS had correctly identified as surgical. This is reflected in the negative predictive value, which rose from only 45.8% with TLICS—implying that nearly half of those recommended for conservative treatment were at risk of a missed unstable injury—to 67.9% (NmTLICS) and 67.7% (CN-mTLICS). By recapturing these missed cases, both modifications reduce the risk of neurological deterioration, progressive deformity, and chronic back pain arising from undertreatment.

Beyond their measured performance, the three systems differ in what they score and how they weight it (Table 6): TLICS grades fracture morphology, neurological status, and PLC integrity; NmTLICS adds objective morphological modifiers (vertebral height loss or canal compromise); and CN-mTLICS further incorporates intervertebral-disc injury while re-weighting the PLC. Severe vertebral collapse and severe disc injury frequently coexist (20), and both are sensitive markers of an unstable burst fracture. Although the overall diagnostic performance of NmTLICS and CN-mTLICS did not differ significantly, this equivalence does not make them interchangeable. Discrepancy analysis was revealing: NmTLICS identified 6 surgical patients missed by CN-mTLICS (those with >50% vertebral compression but only mild disc injury), whereas CN-mTLICS captured 5 missed by NmTLICS (those with severe disc injury but relatively preserved vertebral morphology). Thus, despite comparable aggregate accuracy, the two modifications interrogate different facets of spinal injury and carry genuinely complementary value.

**Table 6:** Conceptual and structural comparison of TLICS, NmTLICS, and CN-mTLICS.

**Dimension**	**TLICS**	**NmTLICS**	**CN-mTLICS**
Fracture morphology	Compression 1/Burst 2/Translation-rotation 3/Distraction 4	TLICS scale + extra 2 points when vertebral height loss >50% or canal stenosis >50%	Same morphology scale as TLICS
Neurological status	Intact 0/Nerve root 2/Complete cord 2/Incomplete cord 3/Cauda equina 3	Identical to TLICS	Identical to TLICS
PLC integrity	Intact 0/Indeterminate 2/Disrupted 3	Identical to TLICS	Intact 0/Indeterminate 1/Disrupted 2
Intervertebral disc injury	Not assessed	Not assessed	Graded 0–2 via MRI using the Sander classification
Surgical threshold	Total score >4 suggests surgery	Total score >4 suggests surgery	Total score >4 suggests surgery
Core innovation	First quantitative classification combining morphology, PLC, and neurological status	Captures severe burst-fracture morphology missed by TLICS	Adds intervertebral disc injury as an independent scoring dimension
Primary limitation	Underestimates severe burst fractures without neurological deficit	Still does not score disc injury	MRI dependence may limit acute-phase applicability

A key strength of NmTLICS is that it can be completed from CT alone, which confers high applicability and timeliness. Because osseous changes such as severe anterior-column height loss and canal encroachment are readily measured on CT, NmTLICS permits a rapid initial judgment of mechanical stability in the emergency setting, without awaiting a time-consuming MRI. This efficient screening reduces examination time and cost and avoids the risk of secondary neurological injury from repeatedly transferring an acutely injured patient for further imaging (21), aligning well with the need for rapid decisions in severe trauma.

However, CT cannot reliably reveal occult soft-tissue injury (22), and reliance on it alone can mislead treatment planning. CN-mTLICS addresses the TLICS blind spot for disc injury by grading the integrity of the disc and endplate on MRI. As Lu et al. (20) emphasized, disc injury and degeneration are closely linked to vertebral fracture and are key drivers of late vertebral collapse and segmental kyphosis, whereas chronic discogenic pain is a common long-term sequela of disc injury (23). By incorporating MRI, CN-mTLICS trades some speed and economy for a prospective read on long-term biomechanical stability, identifying occult risks of late functional decline and informing strategies that balance immediate stability against long-term outcome. In short, NmTLICS functions as an “acute-phase alarm” for immediate osseous failure that CT can trigger quickly, whereas CN-mTLICS offers a “prognostic lens” oriented toward long-term quality of life; used together, they provide a more complete assessment.

For any new classification, inter-observer agreement is a key index of usability and generalizability. It is often assumed that adding morphological variables or subtypes increases complexity and erodes agreement (24); our data show the opposite. Inter-observer agreement was higher for NmTLICS (*κ* = 0.896) and CN-mTLICS (*κ* = 0.866) than for TLICS (*κ* = 0.849). Interpretation of PLC injury under TLICS depends heavily on subjective experience, long recognized as a major source of inconsistency (25). By contrast, NmTLICS introduces objective, measurement-based modifiers (vertebral height loss >50% or canal stenosis >50%), whose explicit quantitative thresholds reduce subjective ambiguity while still capturing injury severity, thereby improving reliability. CN-mTLICS likewise sustains high agreement through clear, well-defined imaging criteria for traumatic disc injury.

For diagnostic tools, greater sensitivity is often won at the expense of specificity, raising the legitimate concern that a more sensitive system might encourage over-treatment. Our data argue against this: only 2 patients were misclassified by NmTLICS, and the specificity of both modified systems did not differ significantly from that of TLICS (*P* > 0.05). Notably, although the modified systems enlarged the decision “gray zone” (total score = 4), this did not blur clinical decision-making; on the contrary, the positive predictive value within this zone remained near 90%, confirming that patients entering it carry genuine surgical indications. Both modified systems therefore refine the scoring structure to capture latent instability without indiscriminately broadening surgical indications, preserving a precision comparable to TLICS.

## 5 Limitations

This study has several limitations. First, actual surgical decision-making was used as the reference standard; however, the treating physicians were not blinded to imaging features that overlapped with components of the scoring systems, such as PLC integrity and intervertebral disc injury, which may have introduced circular-reasoning bias. This reference standard therefore reflects expert clinical judgment more closely than objective patient outcomes. Future validation studies should use patient-centered outcomes as anchor measures, including 12-month VAS, ODI, reoperation rate, and return-to-work rate, to establish a more reliable outcome-based gold standard.

Second, owing to the retrospective design, standardized scale-based assessments were not performed during follow-up, and patient-reported outcome measures (PROMs)—including VAS for back pain, ODI for disability, and SF-36 for quality of life at 6 and 12 months—were unavailable. This study could therefore not determine whether the higher diagnostic accuracy of the modified scoring systems translates into long-term pain relief, functional improvement, or quality-of-life benefits. A prospective cohort extension is currently being designed to collect long-term functional outcomes and further address these questions.

Third, this study was conducted at a single tertiary trauma center, and its generalizability may be limited because case mix, surgical thresholds, and imaging resources may differ from those in community hospitals or primary care institutions. External validation through multicenter prospective registry studies, preferably following the TRIPOD reporting guidelines, is warranted before broad recommendation of either scoring system. In addition, the limited sample size, particularly the small number of nonoperatively treated patients, reduced the statistical power. Although the AUCs of the two modified systems were numerically higher than that of TLICS, these differences in discriminative performance did not reach statistical significance after correction for multiple comparisons. Therefore, the present findings should be regarded as exploratory.

## 6 Conclusion

In this single-center retrospective cohort, the conventional TLICS system may underestimate severe thoracolumbar fractures without neurological deficits. By incorporating objective morphological factors, NmTLICS and CN-mTLICS improved the sensitivity and negative predictive value for actual treatment decisions without markedly reducing specificity, while maintaining good interobserver agreement. Although the AUCs of the two modified systems were numerically higher than that of TLICS, the differences did not reach statistical significance after correction for multiple comparisons. Their advantage should therefore be interpreted as improved case classification around the surgical threshold rather than a significant improvement in overall discriminative performance.

From a clinical perspective, NmTLICS may serve as a CT-based rapid screening tool for assessing osseous instability in the acute phase, whereas CN-mTLICS may provide further evaluation of disc–ligamentous complex injury when MRI is readily available. The stratified and complementary use of these two systems may help balance diagnostic accuracy with clinical feasibility; however, their practical value requires further validation through multicenter prospective studies and health economic evaluations.

## Data Availability

The raw data supporting the conclusions of this article will be made available by the authors, without undue reservation.
